# The exposure-outcome relationship between physical and psychosocial factors and non-specific back pain in adolescents: a systematic review and meta-analysis protocol

**DOI:** 10.3389/fped.2025.1712074

**Published:** 2026-01-12

**Authors:** Gauri A. Oka, Ashish S. Ranade, Rupeshkumar B. Deshmukh, Mayur K. Shinde, Prasad D. Pore

**Affiliations:** 1 Central Research and Publication Unit, Bharati Vidyapeeth (Deemed to be University) Medical College, Pune, India; 2 Blooming Buds Center for Pediatric Orthopedics, Deenanath Mangeshkar Hospital and Research Centre, Pune, India

**Keywords:** adolescents, exposures, non-specific back pain, physical, pooled prevalence, psychosocial

## Abstract

**Background:**

Non-specific back pain in adolescents is widely prevalent, with potential for chronicity in adulthood. Evidence points to various physical and psychosocial exposures of non-specific back pain in adolescents. Reviewing and synthesizing this evidence will help identify modifiable and non-modifiable exposures that can inform appropriate interventions. This systematic review and meta-analysis aims to estimate the global pooled prevalence of back pain in the age group of 10–19 years and describe the exposure-outcome relationships between various risk factors and non-specific back pain. We also aim to establish the dose-response relationship, if possible, between factors such as screen time exposure, BMI, psychological assessment scores, and non-specific back pain.

**Methods:**

A robust search strategy will be employed to search PubMed, PMC, and Scopus databases for articles in English. Observational studies that estimate the prevalence and severity of non-specific back pain in adolescents (age 10–19 years) will be included. Studies that have looked at exposures such as, but not limited to, the mode of transport to school, school bag weight, BMI, screen time exposure duration, physical activity, participants’ perceptions, and psychological, familial, and social factors will be eligible. Two authors will independently screen articles for suitability using the Rayyan software, extract data using the Review Manager 5 software, and assess the risk of bias using the JBI tool for critical appraisal of observational studies. The global pooled prevalence will be calculated using the Freeman-Tukey transformed proportions in the common effect inverse variance model. In case of low heterogeneity (*I*^2^ < 50%), the fixed effect model will be used, and a random effects model, otherwise. Subgroup analyses will be employed for socioeconomic strata, geography, use of school bags, type of school, screen time exposure, frequency and duration of physical activity, and objective assessments on psychosocial evaluation. If data are available, dose-response relationships will be determined between school bag weight, screen time duration, and severity of back pain.

**Discussion:**

Identifying modifiable exposures of non-specific back pain can inform intervention strategies suitable for the individual or school setting.

**Systematic review registration:**

PROSPERO CRD42024572590.

## Background

Back pain is considered non-specific in the event of a normal clinical examination or the absence of a radiological or biochemical cause (1). Many studies conducted across the globe have reported a range of prevalences of back pain in children and adolescents: between 5% and 75% (2, 3). Given the significant proportion of adolescents affected and since back pain in this age group can lead to chronicity of back pain in adulthood (1, 2, 4, 5), it becomes an important public health problem warranting the attention of multidisciplinary stakeholders.

Studies have implicated the weight of the school bag (6–10), poor ergonomics and postural habits (11–13), lack of physical activity (14), or, at times, an excess of it (15), as well as excessive screen time exposure durations (14, 16, 17) as important factors contributing to back pain in adolescents. Researchers have also established the contribution of certain psychosocial factors posing a risk to back pain in this age group (8, 13, 14, 18, 19). Some policymakers have recommended reducing the school bag weights to address back pain in school-going adolescents. However, there is no evidence to show that such interventions were effective in reducing the prevalence or intensity of back pain. Although the subject of research on back pain in adolescents has been in vogue for many years now, the global pooled prevalence of non-specific back pain in adolescents is unknown.

There has been no conclusive evidence regarding the exposures associated with back pain and, if present, the strength of their association with back pain. It also remains unclear whether there is a dose-response relationship between the severity of back pain and certain factors, such as the weight of the school bag, the scores on psychological assessments, or the screen time exposure durations. The following three are the objectives of this systematic review and meta-analysis: 1) to estimate the global pooled prevalence of non-specific back pain in adolescents, 2) to determine the physical and psychosocial risk factors of non-specific back pain in adolescents, and 3) to determine the exposure-outcome relationship between physical and psychosocial factors and non-specific back pain in adolescents.

A synthesis of such evidence will help identify both modifiable and non-modifiable exposures associated with non-specific back pain in adolescents and help devise community and individual-level interventions to tackle the problem. To our knowledge, there is no systematic review and meta-analysis that addresses these questions.

## Methods

The systematic review and meta-analysis protocol is written in compliance with the Preferred Reporting Items for Systematic reviews and Meta-Analyses—Protocols (PRISMA-P) checklist (2015). This protocol is registered in the International Prospective Register of Systematic Reviews (PROSPERO, registration number CRD42024572590).

### Eligibility criteria

Studies will be included if the participants are adolescent girls and boys (aged between 10 and 19 years), regardless of their geographic and socioeconomic settings. Descriptive and analytical observational studies describing the prevalence of non-specific back pain and the physical and psychosocial risk factors of non-specific back pain in adolescents and/or the exposure-outcome relationship between physical and psychosocial factors and non-specific back pain in this age group will be included. Non-specific back pain is defined as pain felt in any area between the first cervical vertebra and the gluteal fold, which cannot be attributed to any specific clinical or radiological cause. The physical factors of interest include, but are not limited to the weight of the school bag, the weight of the school bag as a percentage of the adolescent's body weight, the adolescent's BMI, the mode of transport to school, the method of carrying the school bag, daily screen time exposure, and duration spent engaged in active sports activities. The psychosocial factors include the presence of a family member complaining of back pain, the participant's perceived cause of back pain, and scores on various psychological domains such as emotional, conduct, hyperactivity, peer problems, and prosocial behavior assessed using standard and validated tools.

### Information sources

A detailed search for relevant literature will be performed on PubMed, PMC, Scopus and Google Scholar databases. Due to a lack of free access to databases such as Web of Science, Cochrane Library, and Embase, we have restricted our search to the freely available databases at our institute. However, every effort will be made to collaborate with researchers from institutes with access to paid databases so as to minimize the risk of missing eligible studies. Only English language abstracts and full-text articles will be eligible for screening. Case reports, commentaries, editorials, correspondence, book chapters, and conference proceedings will be excluded.

### Search strategy

A comprehensive search strategy is developed to enable the inclusion of a large number of relevant publications on the topic. Keywords including, but not restricted to, “back pain,” “proportion,” “prevalence,” “epidemiology,” “adolescent,” and “school” will be incorporated into the search strategy. The search will include MeSH terms, appropriate Boolean operators, and truncations where necessary. The search for published literature will be restricted to the time frame between 1st January 2015 and 31st December 2024. The references cited by the selected publications will also be screened for potential eligibility and inclusion.

### Study selection

The results of the search strategy will be imported into the Rayyan software. Predefined inclusion and exclusion criteria as described above will be adhered to decide the eligibility of the studies. Two reviewers (GO and AR) will initially screen the titles and abstracts of the studies independently using Rayyan. Each reviewer will classify the articles into one of three categories, namely, “include,” “maybe,” and “exclude,” while remaining blinded to each other's decisions. Exact and near-duplicates will be identified and deleted. If an article is excluded, the reasons for the same will be entered into the software. A third reviewer (PP) will be responsible for adjudication and sorting discrepancies, if any. Authors of publications will not be contacted in case of insufficiency or ambiguity. Such studies will be excluded. This will be followed by a review of the full texts of the articles to confirm their inclusion. The Preferred Reporting Items for Systematic Reviews and Meta-Analyses (PRISMA) flow chart will be utilized to depict the selection process. The final systematic review meta-analysis will adhere to the PRISMA 2020 guidelines.

### Data extraction

Data extraction will be done using the Review Manager 5 software. Two reviewers (GO and AR) will use a standard predesigned data extraction form to independently extract the following information from the studies: 1) Publication details (article title, authors, country of origin, publication year, and journal) 2) Study characteristics (study design, sample size, sampling technique, study setting, study period, inclusion and exclusion criteria) 3) Participant characteristics (demographic details, weight, height, BMI, physical and psychosocial variables assessed) 4) Data for subgroup analysis: urban-rural settings, use and no-use of school bags, private and public schools. An initial piloting of the data extraction form will be done to streamline the process and establish consistency and uniformity of data extraction among the reviewers. Once done, the two reviewers will adjudicate the data extraction and sort differences, if any. The third reviewer (PP) will be involved to resolve disputes, as required.

### Risk of bias assessment

Each of the two reviewers (GO and AR) will independently use the Joanna Briggs Institute (JBI) tool (20) for observational studies and ascertain the risk of bias. The third reviewer (PP) will sort discrepancies wherever needed. Based on the assessment using the JBI tool, the risk of bias will be classified as high, low, or unclear for each outcome. Even if one domain is classified in the “high” risk of bias category, the overall risk assessment will be a “high” risk of bias. The risk of bias will be low if all the domains are classified as “low” risk of bias. Even if one domain is in the “unclear” category, the risk of bias will be identified as “unclear”. Risk-of-bias assessments will be depicted graphically for each domain, as well as for the overall evidence.

For both, study selection and the risk-of-bias assessment, we will assess the inter-rater agreement between reviewers using Cohen's kappa coefficient.

### Data synthesis

If at least two studies provide comparable data with respect to the outcomes measured, a meta-analysis will be performed. The Review Manager 5 will be used for analysis. The prevalence of non-specific back pain and odds ratios for various physical and psychosocial exposures will be calculated. For the pooled prevalence of non-specific back pain, we will apply the Freeman-Tukey transformed proportions test using the common effect inverse variance model. We will apply the fixed effect model if the statistical heterogeneity is low, that is, the *I*^2^ < 50% or the *p*-value is greater than 0.05. If found otherwise, a random effects model will be used.

In case of high heterogeneity and if data are available, subgroup analyses will be performed as follows: socioeconomic strata (HIC, LMIC, etc.), geography (urban-rural), use or no use of school bags, type of school (private or public), screen time exposure durations, frequency and durations of physical activity, and the various domain scores obtained or objective assessments made based on psychological evaluation. We will perform sensitivity analysis using the “leave-one-out analysis” approach and by excluding studies having a high risk of bias to evaluate their impact on the overall effect size. This will allow us to determine if a single study has a disproportionate influence on the overall results. The results of this analysis will be presented in a summary table and/or a forest plot. If data are available, dose-response relationships will be determined between school bag weight, screen time duration, and severity of back pain will be assessed using meta-regression models. The study-level covariates will be included to explore heterogeneity between studies. The choice of regression model (linear or non-linear) will depend on the nature of the relationship between the factors and the severity of back pain. Results will be presented graphically using bubble plots.

### Publication bias

Funnel plots will be constructed, and publication bias will be ascertained using Egger's test.

### Grading of evidence

The GRADE Pro (Grading of Recommendations, Assessment, Development, and Evaluations) tool will be employed to grade the confidence in the evidence. Two reviewers (GO and AR) will independently evaluate the quality of evidence for inconsistency (heterogeneity), imprecision (broad confidence intervals), indirectness (non-applicability of evidence), and risk of bias. The quality of evidence thus assessed will be graded as high, moderate, low, or very low. The third reviewer (PP) will resolve discrepancies, if any, between the two reviewers.

## Discussion

A lot of work has been done in the field of non-specific back pain in the adolescent age group. This systematic review and meta-analysis will help in determining the global burden of this health issue. We understand that many studies will have included back pain without specifically addressing non-specific back pain in the adolescent age group. Such studies will be excluded. Also, we expect that a wide range of exposures might be addressed, leading to a high degree of heterogeneity in the variables.

A synthesis of this data will help in the identification of various associated exposures and the categorization of these factors as modifiable and non-modifiable. This evidence will be valuable in better understanding non-specific back pain in adolescents and strategizing appropriate interventions for dealing with the same.

Interventions can be tailored to suit the individual and/or the community (for example, the school) setting. They can further be tested systematically and incorporated on a large scale if proven to be beneficial.
